# Cardiotoxicity following thoracic radiotherapy for lung cancer

**DOI:** 10.1038/s41416-024-02888-0

**Published:** 2024-11-06

**Authors:** Gerard M. Walls, Carmen Bergom, Joshua D. Mitchell, Stacey L. Rentschler, Geoffrey D. Hugo, Pamela P. Samson, Clifford G. Robinson

**Affiliations:** 1 Department of Radiation Oncology, Washington University in St Louis, Saint Louis, MO USA; 2 Patrick Johnston Centre for Cancer Research, Queen’s University Belfast, Belfast, Northern Ireland USA; 3 Siteman Cancer Center, Washington University Medical Campus, Saint Louis, MO USA; 4 Cardio-Oncology Center of Excellence, Washington University in St Louis, St Louis, MO USA; 5 Department of Developmental Biology, Washington University in St Louis, St. Louis, MO USA; 6 Center for Cardiovascular Research, Department of Medicine, Cardiovascular Division, Washington University in St Louis, St. Louis, MO USA

**Keywords:** Radiotherapy, Non-small-cell lung cancer

## Abstract

Radiotherapy is the standard of care treatment for unresectable NSCLC, combined with concurrent chemotherapy and adjuvant immunotherapy. Despite technological advances in radiotherapy planning and delivery, the risk of damage to surrounding thoracic tissues remains high. Cardiac problems, including arrhythmia, heart failure and ischaemic events, occur in 20% of patients with lung cancer who undergo radiotherapy. As survival rates improve incrementally for this cohort, minimising the cardiovascular morbidity of RT is increasingly important. Problematically, the reporting of cardiac endpoints has been poor in thoracic radiotherapy clinical trials, and retrospective studies have been limited by the lack of standardisation of nomenclature and endpoints. How baseline cardiovascular profile and cardiac substructure radiation dose distribution impact the risk of cardiotoxicity is incompletely understood. As Thoracic Oncology departments seek to expand the indications for radiotherapy, and as the patient cohort becomes older and more comorbid, there is a pressing need for cardiotoxicity to be comprehensively characterised with sophisticated oncology, physics and cardio-oncology evaluations. This review synthesises the evidence base for cardiotoxicity in conventional radiotherapy, focusing on lung cancer, including current data, unmet clinical needs, and future scientific directions.

## Background

**Fig. 1:**
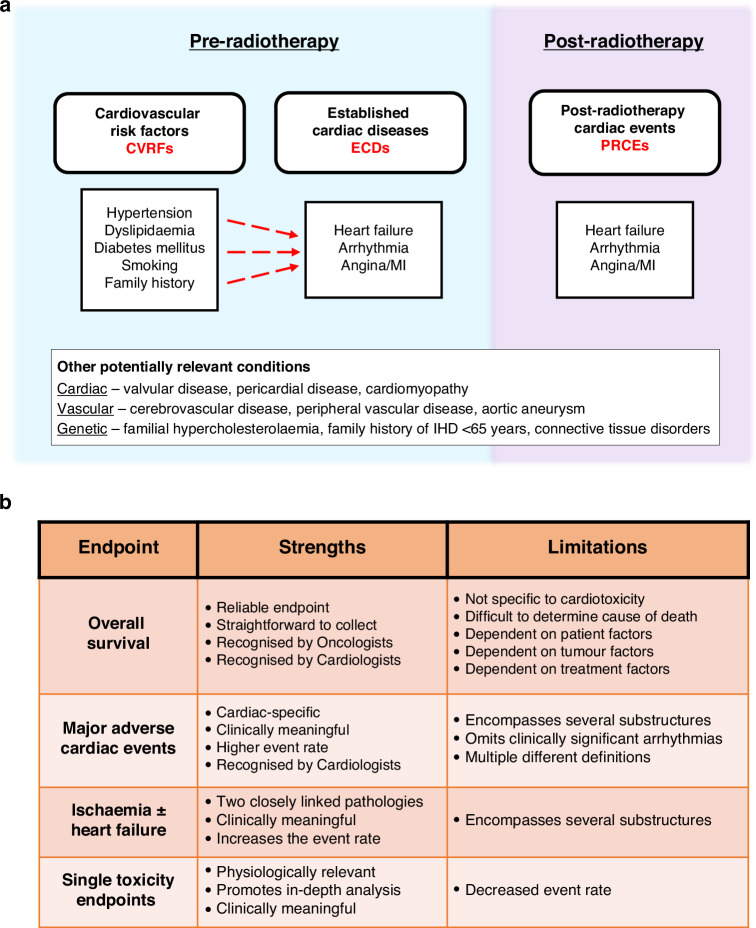
Categorisation of cardiovascular factors and study endpoints in radiation heart disease particularly in the setting of lung cancer. **a** is a summary schema for how critical cardiovascular factors can be considered temporally. **b** displays the positive and negative characteristics of commonly employed study designs. (MI = myocardial infarction; IHD = ischaemic heart disease).

Lung cancer is consistently ranked within the highest causes of death in Western populations [1]. With its high incidence and mortality [2], it is recognised as a ‘cancer of unmet need’ [3]. Definitive radiotherapy (RT) combined with systemic therapy offers the greatest probability of long-term disease control for the high proportion of patients who have potentially curable disease, where surgery is not possible. However, definitive RT carries cardiovascular risk and minimising the morbidity of this treatment is paramount. Cancer control rates from RT are also suboptimal given that 25–40% of patients will experience local relapse [4], yet an increased RT dose led to worse overall survival (OS) in the landmark dose-escalation trial, RTOG-0617 [5]. The elevated mortality rate was associated with higher cardiac radiation doses in this trial. This finding was corroborated by the recent phase 3 LungART trial of postoperative lung RT, where improved disease control with RT likely did not translate into improved OS due to deleterious cardiopulmonary effects [6]. Pooled analyses of historical NSCLC radiation trials examining symptomatic post-RT cardiac events (PRCEs) identified a 2-year cumulative incidence rate of 11–32% [7, 8], consisting largely of arrhythmia, heart failure and ischaemic events. Also relevant is the high prevalence of cardiovascular risk factors (CVRFs) and established cardiac diseases (ECDs) (Fig. 1) before treatment that is typical of lung cancer cohorts [9], owing to considerable tobacco use, lifestyle factors and social deprivation [10]. This review synthesises the evidence base for cardiotoxicity in conventionally fractionated RT specifically and focuses on lung cancer via established research findings, unmet clinical needs, and future directions.

## Cardiotoxicity of radiotherapy: historical context

The first scientific descriptions of radiation-induced heart disease (RIHD) were made in 1924. A German pathologist found extensive cardiac scar formation during the autopsy of a patient previously treated with RT for mediastinal lymphoma who subsequently died from heart failure [11]. A canine experiment investigating radiation-related pulmonary effects in the same year reported fibrotic changes in the atria [12]. However, RIHD was regarded as a rare sequela for a further 4 decades until a case series of breast and lymphoma RT cases implored that the heart was not as radioresistant as previously thought in 1967 [13]. Interest in RIHD then emerged among lung radiation oncologists during 9 adjuvant trials over the following 3 decades [14], although careful attention to minimising heart doses was not embedded in routine practice given the prioritisation of improving dismal cancer control rates in lung cancer, and the lack of technology adequate to do so in most of the older trials.

In 1991 a dedicated interdisciplinary working group in North America made recommendations on the tolerance of normal organs in partial and complete irradiation scenarios [15]. Emami et al. focused on what was considered to be the most severe clinical endpoint for an organ at the time, which was pericarditis for the heart. The authors highlighted that the lymphoma and breast cancer-based recommendations for heart dose constraints, is “…mostly speculative…”, “…from sporadic information in the literature…”, and involved the “…clinical impressions of the clinicians involved…”. These suggested safe dose constraints remained the best available 16 years later, during the design of the RTOG-0617 study. To test the principles of ‘the Emami paper’, the diverse range of dose-fractionations were converted into a common format, based on a radiobiological characteristic, the α/β ratio [16], which animal data suggested was 2.5–3.0 [17–19] for pericardium-related endpoints. The α/β ratio is a summary value for the linear-quadratic behaviour of an organ’s likelihood of developing a specific toxicity [20]. The heart was considered as a single structure with uniform radiosensitivity at the time, as excluding the blood pool from calculations did not alter results [21].

An initiative to summarise the normal tissue toxicity literature two decades after the adoption of three-dimensional RT planning (3DCRT) was unable to make significant advancements for heart constraints, simply concluding that the incidental cardiac dose should be as low as reasonably achievable (ALARA) [22]. The authors noted that lung cancer data requires special consideration, since this patient cohort was not included in prior RIHD analyses and yet had a high rate of PRCEs. The duality of the heart’s radiobiological tissue organisation was also acknowledged, with the left ventricular myocardium possibly being regarded as a ‘parallel’ tissue with respect to muscle contraction, but a ‘serial’ tissue with respect to conduction. Exemplifying the important role of RT technology in RIHD, intensity modulated RT (IMRT), a planning/delivery approach with greater conformality than 3DCRT introduced in the 2000s, has routinely demonstrated lower maximum cardiac doses than 3DCRT [23].

## Cardiotoxicity in clinical trials

RIHD was not truly considered in lung cancer clinics until the RTOG-0617 results were published in 2015. This phase 3, randomised trial in stage III NSCLC found OS to be worse in the high-dose arm (74 Gy) at 20 months versus 29 months in the standard-dose arm (60 Gy) [24]. In a multivariate analysis (*n* = 407) the volume of heart receiving at least 5 Gy (V5) and 30 Gy (V30) were significantly associated with reduced OS, and a secondary analysis indicated V40 was the best heart dose predictor [23]. A retrospective study (*n* = 416) by the RTOG-0617 chief investigators in 2016 corroborated that several heart dose metrics, including V50, are associated with reduced OS, and increased PRCEs, establishing this clinical problem as a new priority [25].

Cardiac outcomes were not specifically embedded as a study endpoint in RTOG-0617, but acute events were captured via standard toxicity assessment. PRCEs occurred in 3% and 9% patients in the standard- and high-dose arms respectively, a rate comparatively lower than published retrospective studies. Ubiquitous conditions such as hypertension and sinus tachycardia were excluded in these calculations, and it is noteworthy that the patients were fitter than the general population as eligibility required Eastern Co-operative Oncology Group (ECOG) status of 0–1. Fatal adverse events were due to cardiac causes in 6 cases, and possible cardiac causes (‘sudden death’, ‘death not otherwise specified’) in 6 cases, although cause of death data was not reviewed by the central trial team. These values may also be underestimates, given the difficulty of determining cause of death in lung cancer [26]. There were considerably more non-protocol contouring deviations for the heart ( ~ 20%) than any other organ-at-risk (OAR) (0–4%) reflecting how cardiac effects were of low concern when the trial activated in 2007 [24]. Furthermore, a breach of the recommended dose limits (V40 < 100%, V45 < 66%, V60 < 33%) was permitted to facilitate any other OAR meeting its threshold.

Another landmark lung RT trial in recent years also exhibited an OS detriment associated with cardiac irradiation. The LungART trial randomised patients with fully resected stage N2 NSCLC to adjuvant RT (heart V30 < 35%) or observation [6]. Grade 3–4 cardiopulmonary events occurred in 11% and 5% of patients, and grade 5 in 8% and 0% for the RT and control arms, respectively. This elevated cardiopulmonary event rate associated with post-operative RT supports the RTOG-0617 conclusion that heart dose is an important treatment consideration, although information on CVRFs and ECDs were also not available for this trial.

RTOG-0617 and LungART provided evidence of significant RIHD and highlight methodological opportunities to advance the field, such as reporting cardiac baseline factors, a range of heart dose metrics and cardiac-specific outcomes. Systematic reviews indicate that cardiotoxicity is under-represented in NSCLC RT trials [27, 28], suggesting some PRCEs are missed [29]. Commercial trials may be particularly prone to over-looking cardiotoxicity, as demonstrated in PACIFIC. This seminal trial integrated immune checkpoint inhibitors with definitive RT for the first time, randomising patients with unresectable stage III NSCLC to 1 year of adjuvant durvalumab or placebo [30]. RT dosimetric objectives were not reported, nor were the heart dose metrics, and no cardiac toxicities were listed in the table of adverse effects [30].

## Retrospective studies

A total of 42 peer-reviewed papers retrospectively examining RIHD in NSCLC have been published, excluding those solely focussed on pericardial endpoints (*n* = 4) given their non-acute and typically asymptomatic nature (Table 1). Only two studies were published prior to RTOG-0617. Approximately 60% of patients were treated with 3DCRT and the rest with IMRT. The RT planning scan type was evenly distributed between 3D and 4D CT, where reported. Dose-fractionation details provided were typically insufficient for calculating the equivalent dose in 2 Gy fractions, and image-guidance details were rarely provided. The mean number of patients and follow-up duration across all studies were 334 (range 43–1190) and 32 months (range 13–74), and patients were treated exclusively within trials in 10 studies.

**Table 1:** Summary characteristics and findings of the peer-reviewed, retrospective, clinical cardiac dosimetry studies in relation to cardiotoxicity.

Author & Year	Cohort Type	Stage	Average EQD2 (Gy)	Follow-Up (months)	Type	*N*	Baseline	Toxicity Endpoint	Planning Constraints	DVHs	MHD	Structures	Atlas	Key Study Findings
Schytte [121]	RWD	I–III	60–80 Gy in 2Gy #*	17.3	3DCRT	250	None	**Primary:** Cardiac events (list included) **Others:** OS	NR	Dmean	NR	LV, bilateral ventricles, heart	NR	15% patients had a cardiac events. DVHs were negative for both events and OS on MVA.
Han [122]	RWD	I–III	60–85.5 Gy in 2–3.8 Gy #*	NR	3DCRT	100	“CVD”, HTN	**Primary:** OS	D33≤66Gy, D66 ≤45Gy, D100 ≤40Gy	Dmax, Vx in 5Gy increments	NR	PA	RTOG	DVHs positive for OS on MVA.
Tucker [36]	RWD	III	60–76 Gy in 1.8–2 Gy#*	24.0	3DCRT (41%) IMRT (49%) IMPT (10%)	468	None	**Primary:** OS	NR	Dmean, V5, V30	16.6	Heart	NR	DVHs negative for OS on MVA.
Speirs [25]	RWD	II–III	50–84.9 Gy in 1.5–2.8 Gy #*	14.5	3DCRT (60%) IMRT (40%)	322	None	**Primary:** Cardiac events **Others:** OS	NR	Dmean, Dmax, Vx in 5Gy increments	2.6 (3DCRT) 1.8 (IMRT)	Heart	Wheatley	24% patients had a grade 3 event. DVHs positive for OS on MVA.
Vivekanandan [33]	Trial	II–III	63–73 Gy in 30#*	25.0	3DCRT (96%) VMAT (4%)	78	Pre-existing ECG only	**Primary:** OS **Others:** ECG change at 6 months	D100 <45Gy, D67 <53Gy, D33 <60%	PCAs	10.3	Pericardium, AVN, 4 chambers, 4 chamber walls	Feng	DVHs positive for OS on MVA. 38% patients had ECG changes. DVHs negative for ECG changes on MVA.
Dess [8]	Trial	II–III	70	23.0	3DCRT (97%) IMRT (3%)	125	HTN, DM, "Pre-existing disease"	**Primary:** G3+ events **Others:** G2+ events, OS	V40 <100%, V65 <33%	Dmean, V5, V30, V50	11.0	Heart	Feng	15% patients had a cardiac event. DVHs positive on MVA. DVHs positive for OS on MVA.
Wang [7]	Trial	III	70–90 Gy in 23-45#*	NR	3DCRT	112	CAD	**Primary:** Symptomatic events **Others:** OS, PCE	V40 <100%, LV V40 <100%	Dmean, V30, V5, LV V30, LV V5	12.0	Heart	Feng	23% patients had a cardiac event. DVHs positive on MVA. DVHs negative for OS on MVA.
Wang [123]	Trial	III	70–90 Gy in 23-45#*	NR	3DCRT	112	CAD	**Primary:** PCE, ACS, arrhyth **Others:** OS	V40 <100%, LV V40 <100%	Dmean, V5, V30, V60	12.0	Feng for chambers, LAD + automated pericardium	Feng	8%, 7%, 11% of patients had a PCE, ACS, and arrhythmia events. DVHs positive on UVA (no MVA). DVHs negative for OS on MVA.
Johnson [124]	RWD	II–III	60–74 Gy in 1.8–2 Gy #*	16.8	3DCRT (38%) IMRT (62%)	86	None	**Primary:** OS	None	V5, V30	NR	Heart	NR	DVHs positive for OS on MVA.
Ma [41]	RWD	I–III	60–76 Gy in 30–38#*	16.9	3DCRT (89%) IMRT (11%)	141	HTN	**Primary:** OS	V30 ≤40-60%, V40 ≤ 30-50%	Dmean, Dmax, Vx in 5Gy increments	5.2	Heart, PA	RTOG	DVHs positive for OS on MVA.
Stam [47]	RWD	II–III	70.1	55.0	IMRT	469	None	**Primary:** OS	Dmax ≤40Gy, D66 ≤50Gy, D33 ≤66Gy	V0.5, V1, V2, V3, V4 and Vx in 5Gy increments & equivalent uniform dose	N/A	Heart	Feng	DVHs positive for OS on MVA.
Guberina [35]	Trial	III	45 Gy in 30# +/− 26 Gy in 13#*	72.0	3DCRT	155	None	**Primary:** OS	NR	Dmean, V5	13.8-17.4	Heart	Feng	DVHs negative for OS on MVA.
McWilliam [61]	RWD	NR	55Gy/20#	NR	3DCRT (NR) /IMRT (NR)	1101 / 89 (SABR)	None	**Primary:** OS	NR	Dmax	NR	Heart base region (not delineated)	N/A	Voxel-based analysis with positive MVA for a dose region approximating to the heart base
Yegya-Raman [40]	RWD	II–IV	60–66 Gy in 1.8–2 Gy #*	47.4	3DCRT (64%) 3DCRT +IMRT (16%) IMRT (19%)	140	CAD, “significant arrhythmia, pericardial disease, HF without CAD, valve disease	**Primary:** Symptomatic events **Others:** OS	V40 <100%, MHD <26Gy as per QUANTEC 2010	Dmean, V5, V30, V50	15.8	4x chambers, LAD	Feng	29% of patients had an event. DVHs positive on MVA. DVHs negative for OS on MVA.
Lee [42]	RWD	III	55–70 Gy in 1.8–2.75 Gy #*	17.6	3DCRT (42%) IMRT/ VMAT (58%)	120	DM, IHD (with definition)	**Primary:** MI **Others:** OS	“QUANTEC” (from 2010)	Dmean, V5, V25, V30, V40, V50, D30	12.6	Heart	RTOG	MI occurred in 4%. DVHs negative for MI on MVA. DVHs positive for OS on MVA.
Lee [125]	RWD	I–III	50–54 Gy in 1.8–2 Gy #*	36.6	3DCRT (70%) IMRT/ VMAT (30%)	43	DM, IHD (with definition)	**Primary:** MI **Others:** OS	Dmean ≤40Gy, V60 ≤33%, V40 ≤80%, V45 ≤60%, V60 ≤33%	Dmean, V5, V25, V30, V40, V50, D30	9.4	Heart	NR	No MI events occurred. DVHs negative for OS on MVA.
Borkenhagen [46]	RWD	I–IV	44–69 Gy in 14–34#*	13.2	NR	76	DM, PVD, "Previous cardiac disease"	**Primary:** Cardiac events **Others:** OS	NR	Dmean, Dmax, V30, V45	N/A	Atria, ventricles, pericardium, coronary space	Wheatley	7%, 21%, 1% of patients had atrial arrhythmia effusions, valve disease. DVHs positive on MVA. DVHs negative for OS on MVA.
Atkins [38]	RWD	II–III	50–66 Gy in 1.8–2 Gy #*	20.4	3DCRT (76%) IMRT (24%)	748	HTN, Lipids, DM, VTE, arrhythmia, valve disease, PVD, stroke, CAD, MI, HF **Primary:** MACE	**Others:**	G3+ events, OS None pre-2008, V30 <50%, V45 <40%, V60 <20% thereafter	Dmean, V5, V30	12.3	Heart	Feng	10% of patients had MACE. DVHs positive on MVA. DVHs positive for OS on MVA.
Hotca [81]	RWD	III	50–80 Gy in 1.8–2 Gy #*	20.0	IMRT	155	DM, DL, HTN, cardiac disease (list included)	**Primary:** ECG Changes	NR	PCAs, Dmin, Dmean, Dmax	NR	Chambers, great vessels	Feng	66%, 35% and 67% patients had an ECG change that was arrhythmic, ischaemic/pericarditic, or non-specific. DVHs positive on UVA (MVA not done).
McWilliam [66]	RWD	III	55 Gy/20#	NR	3DCRT (NR) /IMRT (NR)	978	None	**Primary:** OS	NR	Dmax	NR	Heart base region (not delineated)	N/A	Voxel-based analysis with positive MVA for a dose region approximating to the heart base
McWilliam [62]	RWD	III	55 Gy/20#	NR	3DCRT (NR)/ IMRT (NR)	648	None	**Primary:** OS	NR	Dmax	NR	Right atrium surface (not delineated)	N/A	Surface dose mapping analysis with positive MVA for a dose region approximating to the right atrium
Thor [44]	Trial	III	60 or 74 Gy in 2 Gy #*	24.0	3DCRT (52) / IMRT (48)	306 training / 131 validation	None	**Primary:** OS	Heart V33 <60Gy, V66 <45Gy, V100 <40Gy	Dmean, Dmax, Dmin, MOHX% Dmean, Dmax, and in 5Gy increments	NR	Heart, pericardium, bilateral atria, bilateral ventricles	RTOG	Model combining atria D45, lung Dmean, pericardium MOH55, and ventricles MOH5.
Jang [126]	RWD	III	50–72 Gy/25–36#	27.5	3DCRT (NR)/ IMRT (NR)	258	HTN, DM, CV disease	**Primary:** OS	NR	Mean, V5, V10, V20, V30, V40, V50, and V60	NR	WH, RA, LA, RV, LV	NR	MVA positive for LV in patients with cardiac history only
Vivekanandan [39]	RWD	I–III	69	38.0	3DCRT (75%) VMAT (25%)	64	"Baseline cardiac comorbidity"	**Primary:** OS	NR	Dmean, Vx in multiple thresholds	7.6	Heart, LA	Feng	DVHs positive for OS on MVA.
Sheperd [127]	RWD	I–III	45–70 Gy in 1.8–2 Gy#*	64.0	3DCRT (30%) IMRT (70%)	284	None	**Primary:** OS	V30 ≤50%	Dmean, Dmin, Dmax plus Vx in 5Gy increments	11.2	Heart	RTOG	DVHs positive for OS on MVA.
Xu [97]	Trial	II–IV	60–74 Gy in 2 Gy # *	26.2	IMRT (61%)/ Protons (39%)	225	“Pre-existing heart disease”	**Primary:** Cardiac events **Others:** OS	V30 <50%, V45 <40%, V60 <20%	Dmean, Dmax, Vx in 5Gy increments	12.0	Heart, pericardium, chambers	RTOG	25% of patients had a cardiac event. DVHs not positive for events on UVA. (No MVA). DVHs not positive for OS on UVA. (No MVA).
Atkins [73]	RWD	II–III	50–66 Gy in 1.8–2 Gy #*	NR	3DCRT (76%) IMRT (24%)	701	HTN, Lipids, DM, VTE, arrhythmia, valve disease, PVD, stroke, CAD, MI, HF **Primary:** MACE	**Others:**	G3+ events, OS None pre-2008, V30 <50%, V45 <40%, V60 <20% thereafter	Dmean, Dmax, Vx in 5Gy incre(26)ments	12.3	Chambers & coronaries	Feng	DVHs positive for both MACE and OS.
Niska [128]	RWD	III	43.1–74 Gy in 1.8–2 Gy #*	18.0	3DCRT (41%) / IMRT (59%)	119	None	**Primary:** OS	Dmax <62Gy, Dmean <26Gy, V30 <46%, V40 <33%	Vx in 5Gy increments	NR	Heart	NR	DVHs positive for OS on MVA.
McKenzie [129]	Trial	III	60 Gy in 30# or 74 Gy in 37#*	22.9	3DCRT (54%) IMRT (46%)	439	None	**Primary:** OS	None	Dmean, LAD V15	NR	Heart, LAD	Feng	DVHs positive for OS.
Yu [130]	RWD	III	50–72 Gy in 20–36# (IMPT) or 45–66 Gy in 15–33#*	25.5	IMPT (21%) IMRT/ VMAT (79%)	163	HTN, CAD, DM	**Primary:** Cardiac events	NR	Dmean, Dmax, V5, V10, V20, V30, V40,	3 (IMPT)/ 9 (IMRT)	Heart	NR	12% of patients had an event after IMRT/VMAT, 0% after IMPT.
Cho [131]	RWD	III	60–66 Gy in 2–2.4 Gy #*	45.0	3DCRT (83%) / 3DCRT +IMRT (3%)/ IMRT (14%)	133	Pre-existing heart disease (list included)	**Primary:** G2+ cardiac events	None mandatory	Dmean, V5, V30, V50	8.3	Heart, LV wall	NR	32% patients had a cardiac event. DVHs positive for events on MVA.
Craddock [65]	Trial	II–III	60–74 Gy/30–37#	NR	3DCRT (NR)/ IMRT (NR)	172	LVEF	**Primary:** OS	V40 ≤50%	Dmax	NR	Heart base region (not delineated)	N/A	Voxel-based analysis with positive MVA for a dose region approximating to the heart base
Kim [54]	RWD	I–III	60–64.5 Gy in 30#*	36.2	3DCRT (34%) IMRT (66%)	321	BMI, DM, AF, valve disease, CAD, CHB, stroke, PVD, CAC, alcohol	**Primary:** Cardiac events **Others:** OS	Dmean <45Gy	Dmean, Dmax, Vx in 5Gy increments	12.3	Chambers, coronaries, conduction nodes	Feng, Loap	5% of patients had AF. 2% had non-AF events. DVHs positive for AF on MVA. DVHs positive for OS on MVA.
Banfill [72]	RWD	I–IV	>45 Gy in 20#*	NR	NR	967	HTN, IHD, Myocardial, Pericardial & Valve disease, Arrhythmia (list included)	**Primary:** Cardiac death	V30 <40%, V40 <30% (from 2015)	Dmean, V5, V30, V50,	12.8	Heart	UK SABR Consortium Guidance	DVHs positive for OS on MVA.
No [34]	RWD	IIB–IIIC	NR	74	3DCRT (<1%)/ IMRT (25%)/ VMAT (75%)	233	DM, HTN, previous CV event	**Primary:** Myocardial, conductive, constrictive, valvular	NR	Mean, V7, V15, V27	9.3	WH, LV, LMC, RCA, LCX, LAD, Total left coronary arteries	Duane	22% patients had a cardiac event. Total left associated with cardiac events on MVA
McWilliam [64]	Trial	III	60–74 Gy/30–37#	24	3DCRT (NR)/ IMRT (NR)	458	None	**Primary:** OS	V40 <100%, V45 <66%, V60 <33%	V5, V30	NR	Heart and heart base region (not delineated)	NR	Voxel-based analysis with positive MVA for a dose region approximating to the heart base
Yegya-Raman [111]	RWD	II–IV	66 Gy/33#	29.9	3DCRT (11%) / IMRT (36%)/ proton (57%)	187 / 140	Cardiac comorbidity, CAD	**Primary:** Death without progression **Secondary:** OS	MHD <26Gy	Mean, V5, V30, V50	8.1	Heart,	Feng	Ventricle metrics MVA positive for DWP and OS.
Yegya-Raman [132]	RWD	II–IIIC	66–70 Gy/33–35#	39.6	3DCRT (6%) / IMRT (59%)/ proton (35%)	335	HTN, DM, Lipids, previous atrial arrhythmia	**Primary:** MACE **Secondary:** Cardiac events, OS	MHD <20Gy, V50 <25%	Mean, Dmin, Dmax, V5-70	8.7	Heart, LV, LAD	Feng	10.4% patients had a MACE. MVA positive for OS only, for MHD and LAD V15
Olloni [133]	RWD	NR	24–33 #	NR	3DCRT (9%) / IMRT (41%)/ VMAT (50%)	644	Arrhythmia and “baseline heart disease”	**Primary:** OS	NR	V2 – V70 (2Gy intervals)	11.3	WH, RA, LA, RV, LV, LMC, LCX, LAD, RCA	NR (auto)	MVA positive for OS (2 x PCAs)
Walls [134]	RWD	I–III	55–84 Gy/20–42#	NR	3DCRT (29%) / IMRT (20%)/ VMAT (51%)	478	HTN, DM, Lipids, previous MI, previous HF, previous arrhythmia	**Primary:** OS **Secondary:** MI, HF, arrhythmia	MHD <26Gy	Dmax	NR	Heart base region	Christie Definition	17% patients had a cardiac event. MVA positive for both endpoints
Walls [57]	RWD	I–III	55 Gy/20#	NR	3DCRT (20%)/ IMRT (30%)/ VMAT (50%)	420	HTN, DM, Lipids, previous MI, previous HF, previous arrhythmia	**Primary:** AF	MHD <26Gy	Mean, V5, V55	NR	Pulmonary veins	Walls	6% patients developed AF. MVA positive
Atkins [135]	RWD	II–III	50–66 Gy in 1.8–2 Gy #*	20.4	3DCRT (76%) IMRT (24%)	748	HTN, Lipids, DM, VTE, arrhythmia, valve disease, PVD, stroke, CAD, MI, HF	**Primary:** G3 arrhythmia subtypes	None pre-2008, V30 <50%, V45 <40%, V60 <20% thereafter	Dmean, Dmax, Vx in 5Gy increments	12.3	Chambers, coronaries, PVs, nodes	Feng	17% experienced ≥1 G3 arrhythmia. On MVA analyses, PVs associated with AF and SVT, LMC with VT/asystole, LCX with atrial flutter, and the RCA with bradyarrhythmia

*(N = number of patients; EQD2 = equivalent dose in 2 Gy fractions; NR = not reported; DVH = dose-volume histogram; MHD = mean heart dose; 3DCRT = three-dimensional conformal radiotherapy; IMRT = intensity-modulated radiotherapy; HTN = hypertension; DM = diabetes mellitus; OS = overall survival; MVA = multivariate analysis; PCE = pericardial event; ACS = acute coronary syndrome; LAD = left anterior descending coronary artery; UVA = univariate analysis; DL = dyslipidaemia; VTE = venous thromboembolism; PVD = peripheral vascular disease; CAD = coronary artery disease; MI = myocardial infarction; HF = heart failure; MACE = major adverse cardiac event; RTOG = Radiation Therapy Oncology Group; IMPT = intensity-modulated proton therapy; ECG = electrocardiogram; AVN = atrioventricular node; CVD = cardiovascular disease; PA = pulmonary artery; LV = left ventricle; RV = right ventricle; LA = left atrium; RA = right atrium; RCA = right coronary artery; LMC = left main stem coronary artery; LCX = left circumflex coronary artery; IHD = ischaemic heart disease; BMI = body mass index; AF = atrial fibrillation; CHB = complete heart block; CAC = coronary artery calcification; SABR = stereotactic ablative radiotherapy; DWP = death without progression; VBA = voxel-based analysis; MOHX%[Gy] = mean of the hottest x% of the volume, in Grey; LVEF = left ventricular ejection fraction)*

(* = EQD2 not reported, so physical prescription provided)

The primary endpoint was cardiac events for 17/42 studies and OS in 20/42 studies, with other endpoints including electrocardiogram (ECG) changes (Table 1). Only a minority of studies reported baseline cardiac status, i.e. CVRFs (e.g. hypertension, dyslipidaemia, diabetes mellitus, smoking, family history) and ECDs (e.g. arrhythmia, heart failure (HF) and acute coronary syndrome (ACS)). Cardiovascular risk scores were provided in only 10/42 studies.

A diverse range of cardiac dose constraints were used during treatment planning, and where these were reported, they were for the whole heart (WH). Hearts were generally contoured based on the Feng atlas [31] (*n* = 15/42) or Radiation Therapy Oncology Group guidance [32] (*n* = 6/42) (Table 1). Sixteen studies delineated at least 1 cardiac chamber, and at least 1 coronary artery was contoured in 7 studies. Chamber walls, without the intraluminal blood pool, were segmented in 1 study [33]. The most common dose volume histogram (DVH) summary metrics employed were the mean dose (Dmean) and maximum dose (Dmax).

The large range of average mean heart doses (MHD) among patients in the cohorts, 2.2–16.6 Gy, epitomises two decades of RT technology evolution, the large variability in the anatomic relationship of the lung and involved nodes relative to the heart, and global dose-fractionation prescription diversity (Table 1). The mean incidence of pooled PRCEs was 19.7% (range 7–32%) with an average follow-up of 26.0 months (range 13.2–45.0). Of the 14 studies where a multivariate analysis was performed for a dosimetric parameter as a predictor of PRCEs, 10 found a statistically significant association. Using OS as a surrogate endpoint for RIHD, 30 of 38 studies found statistically significant associations with at least one DVH metric on multivariate analysis. Only one study included patients that underwent curative-intent re-irradiation [34].

**Fig. 2:**
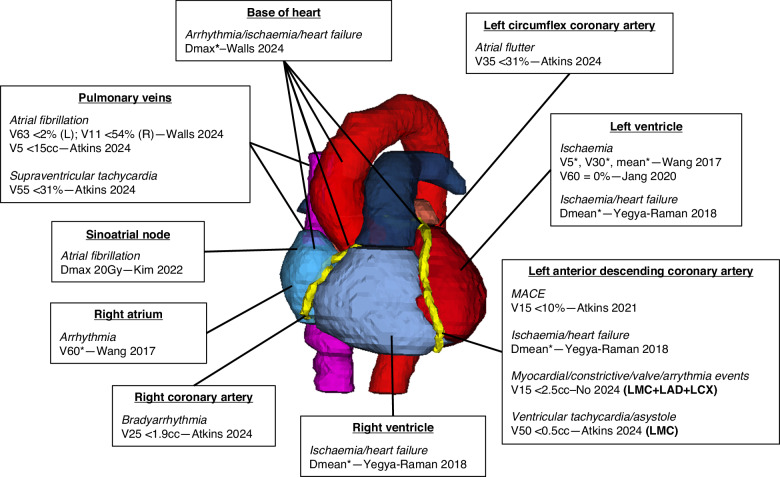
Summary of published dosimetric relationships for cardiac endpoints in lung radiotherapy studies. The three-dimensional reconstruction of autosegmented cardiac substructures from a lung cancer radiotherapy plan, annotated with the literature on post-radiotherapy cardiac event endpoints by substructure, and proposed dose constraints where available (*no dose threshold reported; Vxx = percentage volume receiving at least XX Gy; Dmax = maximum dose; Dmean = mean dose).

In these retrospective analyses of heterogenous patients with varying degrees of statistical rigour, the heart V5 and V30 flagged in RTOG-0617 were not validated for OS in all datasets [7, 35–37]. While other metrics such as MHD did show utility in several cohorts [38, 39], this was not a consistent trend [7, 8, 40–42], aligning with a systematic review that found no consistent relationship between any WH metric and OS or cardiac outcomes [43]. Several studies failed to find a relationship between heart V5 and V30 and PRCEs [7, 8, 37], prompting deeper analyses with cardiac substructures [44]. Supervised analysis of these distinct regions may be viewed as a systematic and biologically refined approach. Some of the key NSCLC literature on cardiac substructure are summarised in Fig. 2.

## Optimal endpoints

In theory, optimal endpoints for RIHD studies would include PRCEs, cardiac mortality, and all-cause mortality, which can help capture the competing effects of cancer-specific mortality and mortality from radiation side effects including RIHD. In practice, assessing and interpreting these endpoints across studies is challenging due to heterogenous definitions. Some studies record all PRCEs that are symptomatic, others use composite cardiology endpoints, while others choose oncological toxicity criteria (e.g. Common Terminology Criteria for Adverse Events), which requires careful interpretation (Fig. 1) [45]. Interpreting grade 3 PRCEs (e.g. hospitalisation) can be problematic, since the criteria for an inpatient admission varies greatly between healthcare systems. It is also worth noting that due to their chronic implications, many grade 1–2 PRCEs generate the requirement for ongoing for primary care interactions and subsequent investigations, which can also impact quality of life via time toxicity and polypharmacy and therefore are likely to negatively impact survivorship. Furthermore, patients that develop low-grade PRCEs may do so because of better health at baseline, and therefore including only patients with grade 3 events may inadvertently introduce bias.

Importantly, only a minority of studies in NSCLC have found an association between PRCEs and death [8, 40, 46], suggesting historically reported PRCEs have not been tightly coupled with cardiotoxicity-related deaths. Although death events are regarded as a reliable endpoint in oncology, they are an imperfect endpoint in RIHD for patients with NSCLC. Investigating the precise mechanisms by which cardiac dose leads to death is complicated by the central role played by the heart in the systemic response to acute medical conditions, leading to difficulty in accurately discerning the cause of death for patients with lung cancer [26]. The absence of sensitive post-RT cardiac surveillance tests means occult disease may go undetected. It has been posited that non-event (subclinical) cardiotoxicity is not clinically important until the onset of an acute medical stressor such as sepsis or haemorrhage, where it manifests to hamper recovery, increasing the risk of death from the intercurrent medical problem [47–49]. Supporting this hypothesis, post-RT cardiac magnetic resonance imaging exhibits cardiac parenchymal changes in asymptomatic patients [50, 51]. Furthermore, the low rate of fatal PRCEs reported in the literature does not fully account for the increased death rate observed with higher heart doses in NSCLC, positing subclinical cardiac insults as a vector for increased mortality. As such, all-cause mortality may serve as a surrogate metric for RIHD in large cohorts where relevant patient, tumour and treatment data are available. In practice, the magnitude of the cardiac event rate, excess death rate and quality of life detriments [52] equally justify the urgent formulation of cardioprotection principles. That cardiac dose may also behave as a surrogate for other toxicity endpoints such as pulmonary is also under investigation [53].

## Analytical challenges in radiation cardiotoxicity studies

There is a wide range of methods available for elucidating the relationship between radiation dose and toxicity, known as normal tissue complication probability modelling, and there is no consensus on the optimal approach. While multivariable regression models typically comprise the definitive statistical test of these studies, there is great variability among the processes by which substructures, dose metrics and thresholds are selected for inclusion. Inter- and intra-substructure dose metric multicollinearity complicates this process, in that some proposed high-risk substructures or critical metrics may actually be a surrogate for an adjacent substructure or a similar metric that has greater influence on the probability of toxicity. For example, a post-RT atrial fibrillation study reported significant associations for the sinoatrial node [54], even though this region of specialised myocardium does not have a clear pathophysiological role in this disease. The investigators employed ‘area under the curve’ values to select candidate dose metrics from a range of almost 150, without any correction for multiple testing [55]. In contrast, a hypothesis-driven study of the pulmonary veins (PVs) [56, 57] found compelling, physiologically sound associations for these structures, and has been validated subsequently [58]. These studies found the relationship of the sinoatrial node to incidence of new atrial fibrillation to be much weaker than that of the PVs, suggesting that the sinoatrial node is merely a surrogate structure, when defined as per Loap [59]. While the use of ‘area under the curve’ values to select candidate metrics may maximise the opportunity to find the ground truth, there is the theoretical cost of a higher risk of deriving a false-positive finding [60].

Contemporary computational methods such as voxel-based analysis may offer an alternative system for identifying critical cardiac regions and their dose thresholds. These high-throughput approaches have been successfully applied to establish the heart base as a general region of interest [61–63] in real-world datasets, with subsequent retrospective validation in clinical trial cohorts [64, 65], and following refinement of the region to a specific composite of anatomically-defined substructures [66].

There is a lack of consensus on how clinical factors that are to be adjusted for in multivariable analysis are selected. Many studies, especially retrospective studies, base these decisions on univariable regression results that meet arbitrary *p* value thresholds, but including known clinically relevant factors may be stronger. It is important to consider the number of events when choosing the number of clinical variables to include in multivariable models, as over-fitting can occur when an excessive amount of variables are included (e.g. 1 variable for every 10 events often used as a general principle).

Lastly, although it is widely acknowledged that inclusion of continuous data is superior to dichotomised data in clinical models, dichotomisation can be useful in RT toxicity studies, since the output may serve as a directly implementable dose constraint for treatment planning. The study design should guide this decision, with continuous data perhaps being more important for ‘discovery’ studies, while dichotomisation maybe more acceptable in studies validating a previously identified metric and threshold. In summary, methodological research is urgently required for the most statistically responsible approaches to be identified.

## Baseline cardiac status

The contribution of baseline cardiac disease relative to dosimetric factors is a current gap in the RIHD literature. The co-morbidity burden of the general lung cancer population is large, with ~30% of patients having a previous ECD [9] and a further 50% having CVRFs that pre-dispose them to future PRCEs [26]. Potentiating this risk, social deprivation in this cohort [67, 68] may lead to suboptimal health service access and engagement, resulting in patients being less likely to have their risk factors optimised [10, 69, 70].

In a study of mixed thoracic cancers where patients had cardiac stents prior to RT, comorbidity burden appeared to drive survival rather than radiation dose parameters [71]. Similarly, the impact of the heart base region dose on survival was mediated by the baseline systolic function in a clinical trial cohort [65]. Cardiac comorbidity was found to be significantly associated with the risk of cardiac death after RT at 2 years (21% versus 6%) in another study [72]. Several of the cardiac substructure studies found a difference in the rate of PRCEs when stratifying by baseline cardiac status, including a 2-year MACE rate of 12% versus 3% for patients with and without cardiac disease [73], and a 4-year incidence of symptomatic cardiac events of 52% versus 23% where there were ECDs at baseline compared to without [40]. The relative risk of PRCEs was lower for patients with ECDs, possibly because of the high rate of PRCEs without significant radiation doses. One interpretation of this is that patients with less baseline cardiac comorbidity should be prioritised for rigorous cardiac substructure avoidance during treatment planning, as the detriment appears to be relatively larger for these patients. However, where such resources are not limited, the high absolute risk of PRCEs in patients with a significant cardiac history, means all patients stand to benefit from these and other cardioprotective strategies.

There is considerable variation between studies regarding the classification of pre-treatment cardiac comorbidities. Some investigators focus on the history of specific cardiac events, such as myocardial infarction (MI) and arrhythmias [72], while others focus on pooled ECDs [73]. As the natural history of cardiovascular disease would dictate that ECDs are end-organ consequences of CVRFs (e.g. hypertension, dyslipidaemia), it is plausible that patients with risk factors but not yet ECDs are also at heightened risk of RIHD. Clinical CV parameters and several CT-based correlates have demonstrated utility for selecting a high-risk group for cardiac assessment and optimisation associated with the incidence of PRCEs but not mortality in the NI-HEART cohort [74]. Recent European cardio-oncology guidance should improve awareness of baseline risk factors by recommending that all patients undergo calculation of a CV risk score [75]. Further recommendation statements could build on this foundation by attempting to harmonise how baseline CVRFs and ECDs are recorded.

Animal data suggests the magnitude of lung irradiation is also relevant when studying RIHD [76], which is logical given these organs are physiologically coupled. However, there is a dearth of clinical data on the impact of baseline pulmonary morbidity. Unfortunately, the inescapable geometric coupling of the heart and lungs means that any sparing of the heart doses may elevate the lung dose with standard photon-based treatments [77, 78]. An ongoing photon versus proton study may address this question [79] and functional lung-sparing RT planning techniques may enable individualised lung dose-sparing [80] in the future if the relationship between lung disease and cardiac post-RT outcomes proves significant when evaluated.

## Investigations for detection of subclinical cardiac injury

In relation to subclinical cardiac dysfunction, ECGs, echocardiograms and other imaging modalities have been tested for assessing asymptomatic toxicity from conventional RT. Among the 78 reportable patients from the IDEAL-CRT trial [33], the proportion of patients with a normal ECG dropped from 49% to 23% within 25 months post-RT, with 14% and 12% patients having new ischaemic/pericardial and rhythm changes, respectively. Furthermore, ECG changes were associated with worse survival. A separate study found that ECG changes were associated with reduced survival [81]. This study had higher rates of new abnormalities (35% ischaemia/pericarditis, 66% arrhythmia) over 20 months’ follow-up. This study also identified an association between higher superior cava minimum radiation dose and new non-specific ECG abnormalities post-RT.

Established imaging techniques from cardiology have also been evaluated for utility in detecting RIHD. Myocardial perfusion imaging has demonstrated that asymptomatic tissue changes are spatially mapped to regions of the highest RT dose distribution [51]. Reductions in cardiac muscle ^18^FDG uptake on tumour response assessment positron-emission tomography/CT scans have also been found after RT and were associated with worse OS [82]. A recent PET-based radiomic analysis identified quantitative imaging features that predict for myocardial SUV changes following RT [83]. Ventricular strain abnormalities have been identified with echocardiogram and are more sensitive than ejection fraction in some RT studies [84, 85]. The European cardio-oncology guidelines recommend baseline echocardiography for patients with an ECD if the heart is within the irradiated volume [75]. However, high quality data to support these recommendations is lacking, and minimum dose criteria were not outlined. While there are no clearly validated CT-based markers of RIHD at present, coronary artery calcification on baseline imaging has been tested semi-quantitatively and quantitatively [34, 74, 86, 87] and was associated with PRCEs in both scenarios.

With respect to circulating markers of RIHD, a wide range of established (e.g. troponin, NT-proBNP) and novel analytes [88] have been investigated, without a clearly superior option being identified (Supplementary Table 2). However, studies have generally consisted of small, mixed tumour cohorts, with a range of dose-fractionations and varied timepoints for blood sampling [89–96], although one group analysed samples from an interventional trial [97]. This study, which compared photon-based IMRT and passive-scattering protons, found that troponin elevations at both baseline and post-RT were associated with PRCEs, irrespective of treatment modality [97]. Overall, that several studies failed to validate signals from animal models reiterates the complex biology at play in humans, in terms of baseline cardiovascular co-morbidity and partial heart radiation exposures.

## Cardiac segmentation

The first peer-reviewed heart atlas was published online in 2010, aimed at breast cancer radiotherapy planning [31]. This atlas not only specified detailed limits for segmentation of the WH, but also for the pericardium and several cardiac substructures. Atlases focussing on specific groups of substructures were subsequently developed, including atlases for the coronary arteries and LV segments [98, 99], the valves [100], conduction system [59, 101] and pulmonary veins [56]. There is duplication between several of the atlases, with varying degrees of congruity. For example, the superior vena cava is excluded from the WH contour in the Feng atlas [31], but included in the Milo atlas [99]. The auricles are included in the Feng atlas images but not described in the text, while Milo recommends including the right but not the left. Additionally, the Duane [98] and Milo atlases use differing landmarks to identify the limits of the coronary artery segments. Some of the published atlases have additional weaknesses. For example, the inter-operator variability for the valves was high in the Socha atlas, and the conduction nodes atlas does not report the process used to derive the definitions. At present, there are no atlases for the great vessels, such as the aorta and pulmonary artery, although it is debated if these vascular structures truly constitute cardiac substructures per se. Nonetheless, substructure-specific data represent a crucial component of RIHD research, given the wide variety of cardiotoxicity subtypes. For example, in relation to arrhythmia, atrial fibrillation, atrial flutter and sinus bradycardia originate in the atria, whereas heart block occurs in the atrioventricular node, and QT prolongation and ventricular tachycardia originate in the ventricles.

Despite the emerging rationale for considering the cardiac substructures during treatment planning, there are a number of barriers to full implementation, such as added delineation time and risk of interobserver variability. The latter issue is secondary to the complexity of substructure geometry, limited intracardiac soft tissue definition, particularly without contrast enhancement, and motion artefacts. Another unresolved issue in cardiac substructure segmentation is whether the cardiac chamber blood pool should be included for the chamber dose calculations. Cardiac substructure autosegmentation tools can fortunately overcome many of these barriers [102].

## Treatment planning

A recent systematic review of published dose constraints noted a myriad of options were included during RT treatment plan optimisation, the most common of which were MHD < 26 Gy (4 studies) and V40 ≤ 30% (3 studies) [103]. Notably, average MHD was not different between IMRT (10.9 Gy) and 3DCRT (10.6 Gy) [103].

Although avoidance of toxicity is important for all organs, the thoracic OARs commonly need to be prioritised for dose-sparing since it is frequently not possible to meet all of the dose constraints. Beyond the long-held belief that many patients with intrathoracic cancers do not survive long enough to experience RIHD, the heart has been given a low priority historically for several other practical reasons. Firstly, cardiotoxicity rates were lower than oesophagitis or pneumonitis. The low-quality evidence base for the putative WH dose constraints also failed to convince some clinicians that negative prognostic correlates of heart dose do not merely reflect unfavourable disease (e.g. node-positive) [104]. Lastly, there have been no globally agreed-upon guidelines for heart delineation. Some definitions include significant portions of the proximal great vessels so that their encapsulating pericardium is included (Supplemental Table 1), despite the fact that symptomatic pericardial problems are infrequent in the era of conformal RT. As well as offering false reassurance that the heart dose is low for many cases, the dose summary values from this version of the WH structure have less relevance for the critical cardiac events. For as long as cardiac doses are assessed on a WH basis in clinical practice, it may be prudent that critical cardiac substructures are reviewed on all CT slices in order to identify mal-placed hot spots, e.g. in the LAD.

For dosimetrists to integrate cardiac substructures in routine clinical workflows, several adaptations of the current RT pathway for NSCLC are needed. Firstly, a high quality simulation, including respiratory management and use of contrast agents where appropriate, is required for the cardiac landmarks to be identifiable. Secondly, more streamlined delineation approaches are needed as this is currently time-consuming, particularly during an operator’s learning phase. Thirdly, putative dose constraints for the optimiser software to attain will be required if plans are to be cardiac-optimised, because the ALARA principle may not be maximally effective for such spatially clustered structures. Because of the small size of the many of these structures relative to the magnitude of respiratory motion, motion envelopes and planning risk volumes will be important.

Several groups have explored the feasibility of incorporating cardiac substructures in silico. One team re-optimised plans for cardiac substructures and achieved significantly lower values for 30 of 32 cardiac substructure dose-volume parameters, using a contemporary version of IMRT known as volumetric modulated arc therapy (VMAT) [105]. While the number of arcs (and treatment time) were increased compared to planning without substructure optimisation, the cardiac-optimised plans did not result in greater doses to other thoracic OARs. Similarly, another arc therapy study demonstrated that introducing a left atrium dose constraint was feasible and could lead to multiple other dosimetric benefits including lower lung doses [106]. Additionally, similar findings were also observed in an MRI-based mixed thoracic cancers planning study [107]. The authors of this paper also reported the cardiac substructure dose objectives utilised for optimisation.

Another priority for radiation physicists is motion management for the cardiac substructures, given their cyclical shape changes, which are irregular in space due to motion from the lungs. As modern RT planning often accounts for tumour motion, a planning volume at risk (PRV) margin for the cardiac substructures may be warranted. PRV margins of 5.8 mm and 4.8 mm were recommended for compensation of WH motion in the lateral and cranio-caudal axes respectively [108]. However, independent displacement of the individual cardiac substructures is likely to be under-estimated by WH margins, as virtually any plane through the heart contains multiple substructures exhibiting non-synchronised and non-isotropic deformation. Furthermore, substructure motion cannot be assumed to be oscillatory after interactions from concurrent respiratory motion are considered. It would be important that adoption of such technical approaches be founded through robust evidence in order to justify the additional resource required, and at present there are no such supportive data, possibly becasue the pragmatic trials required are difficult to design and fund.

## Concomitant systemic anticancer therapies

Another clinically relevant factor is the chemotherapy delivered alongside RT, which is serves to radiosensitise lung tumours, as exemplified by increased survival rates compared to RT without chemotherapy [109]. However, the independent risk of cardiotoxicity from cytotoxic therapies is well established [110]. Unfortunately, many lung radiotherapy studies do not include this factor in multivariable analyses, possibly because the large academic centres involved have very low rates of radiotherapy without chemotherapy, due to the select patient population treated. In those studies where chemotherapy was adjusted for, none reported a significant relationship with cardiac endpoints in multivariable analysis [34, 57, 72, 111]. This trend may be due to the fact that some of the higher risk systemic therapies such as anthracyclines are not typically employed in the management of lung cancer. Interestingly, one group investigated if there was a difference between cisplatin and carboplatin delivered with RT and found the former to have a significant relationship with cardiotoxicity [112]. Only one published study has examined if durvalumab, the adjuvant immunotherapy that has been standard of care after lung cancer RT since 2018, is associated with cardiotoxicity, and the authors found no evidence of a relationship [113]. Given the potential acceleration of atherosclerosis that has been reported with such immune checkpoint-based immunotherapy [114], further study of this factor in future studies continues to be warranted, and novel drugs being trialled as radiotherapy–drug combinations [115] should also be carefully examined.

## Discussion

The heart is arguably one of the most heterogenous OARs, comprised of spatially distinct tissue types with differing radiobiological characteristics, plus functional inter-dependence and geometric instability. Cardiotoxicity is a common adverse effect of definitive RT in patients with lung cancer that results in physical deconditioning, hospitalisation, and non-cancer death during survivorship. RIHD can be distressing for patients with lung cancer, who commonly concurrently deal with symptoms of lung disease, treatment sequelae and the lung cancer itself. For clinicians, RIHD continues to represents a major barrier to treatment intensification as evidenced by the negative dose escalation and acceleration studies.

Thirty years after Emami and colleagues omitted lung cancer data in their landmark papers on cardiac dose-volume characteristics, lung cancer continues to evade rigorous cardiac radiobiological interrogation. Ultimately, a collaborative research effort between translational radiobiologists, cardiovascular academics and radiation oncologists will be required in order to progress the field, so that the clinical goals of prevention, early detection and prompt treatment of RIHD can be met. Issues raised in Emami’s influential paper still hold true for RIHD:

*“Comparative overview of radiation dose tolerance of normal tissue is a major task… The diversity of organs, endless variations in any combination of radiotherapeutic parameters such as fractionation, volume, overall time, etc, physiological status of the organs prior to the commencement of radiation… are some of the factors that makes this task an enormous undertaking.”*

Despite a century of pre-clinical research, there is a severe lack of RIHD experiments in co-morbidity animal models [116]. While many features of cardiac radiopathology have been successfully described in isolation with pre-clinical methods [117], the multitude of inter-connected dose- and time-dependent and spatially-specific pathophysiologies underpinning RHD have not been probed with contemporary biomedical research pipelines. Furthermore, although multiple studies have shown a cardiac radioprotective effect for several drugs, none of these have been tested in a clinical trial. Statin therapies have the greatest amount of evidence in this area [118–120] but as most patients with lung cancer meet the criteria for a statin on cardiovascular grounds, conducting a randomised controlled trial is not warranted or feasible. Rather, radiation oncologists are well positioned to take stewardship of such primary prevention therapy when assessing new NSCLC cases. It is also prudent that patients are fully informed of the risk of RIHD in meaningful terms, so that help is sought promptly should they be affected. Active monitoring of select cases following stratification by baseline risk and any added RT-associated risk, or pre-treatment cardiac optimisation, for which the feasibility is being evaluated in the CARMA study (NCT05403736), are expected to significantly improve the cardiac safety of lung cancer RT.

In the future, it is possible that treatment planning will involve enhanced individualised treatment planning goals in order to capitalise on all available baseline risk information, in terms of the tumour location and pre-existing cardiac health. For example, a patient with excellent pulmonary function but severe coronary artery disease and a medial left lower lobe tumour might be permitted to have lung doses slightly above the local constraint to enable the LAD to be spared. Adjustment for cytotoxic, immune checkpoint inhibitors and emerging novel concomitant systemic therapies delivered with RT is also vital.

**Fig. 3:**
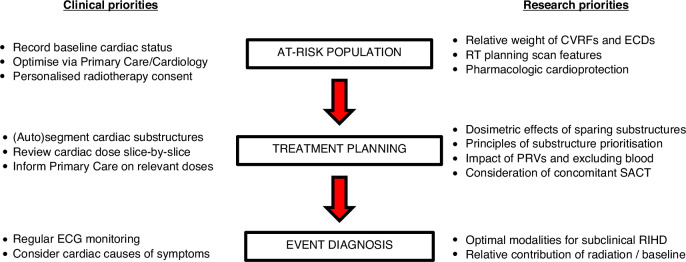
Suggested priorities at each stage of the patient journey for both clinicians and researchers. (CVRF = cardiovascular risk factor; ECD = established cardiac disease; ECG = electrocardiogram; RIHD = radiation-induced heart disease; PRV = planning organ at risk volume).

Suggested current priorities for the clinicians and researchers are summarised in Fig. 3. The RTOG 1308 randomised controlled trial (NCT01993810) comparing photons versus photons in stage III NSCLC has included RIHD within the composite primary endpoint and is likely to provide high-quality data on this topic when it reports in 2026 An international prospective registry of patients with lung cancer undergoing definitive RT would address many of the standardisation issues. Preparatory work will be needed to elicit the most statistically responsible approach to such analyses, in terms of multiple testing and multicollinearity. Ongoing prospective trials particularly CLARITY (NCT04305613) is likely to generate high quality data on how established and experimental blood-, ECG- and imaging-based parameters could be leveraged to identify at-risk patients, and how to actively monitor for toxicity. Robust prospective clinical and biomarker data will serve to inform the design of cardioprotective clinical trials for patients with NSCLC.

## Supplementary information

Supplementary Material
