# Correction: Cardiotoxicity following thoracic radiotherapy for lung cancer

**DOI:** 10.1038/s41416-024-02926-x

**Published:** 2025-01-07

**Authors:** Gerard M. Walls, Carmen Bergom, Joshua D. Mitchell, Stacey L. Rentschler, Geoffrey D. Hugo, Pamela P. Samson, Clifford G. Robinson

**Affiliations:** 1 Department of Radiation Oncology, Washington University in St Louis, Saint Louis, MO USA; 2 Patrick Johnston Centre for Cancer Research, Queen’s University Belfast, Belfast, NI USA; 3 Siteman Cancer Center, Washington University Medical Campus, Saint Louis, MO USA; 4 Cardio-Oncology Center of Excellence, Washington University in St Louis, St Louis, MO USA; 5 Department of Developmental Biology, Washington University in St Louis, St. Louis, MO USA; 6 Center for Cardiovascular Research, Department of Medicine, Cardiovascular Division, Washington University in St Louis, St. Louis, MO USA

**Keywords:** Radiotherapy, Non-small-cell lung cancer

Correction to: *British Journal of Cancer*; 10.1038/s41416-024-02888-0, Published online 06 November 2024

During the proofing process for this article, the authors requested that the rows in Table 1 be sorted by the number present in Column A ‘Author and Year’; this was overlooked by the publisher. Table 1 has been corrected accordingly. Furthermore, the article was mistakenly published with black and white figures, which has also been corrected.

**Table 1:** Summary characteristics and findings of the peer-reviewed, retrospective, clinical cardiac dosimetry studies in relation to cardiotoxicity.

Author & Year	Cohort Type	Stage	Average EQD2 (Gy)	Follow-Up (months)	Type	*N*	Baseline	Toxicity Endpoint	Planning Constraints	DVHs	MHD	Structures	Atlas	Key Study Findings
Schytte 2010 [1]	RWD	I–III	60–80Gy in 2Gy #*	17.3	3DCRT	250	None	**Primary:** Cardiac events (list included) **Others:** OS	NR	Dmean	NR	LV, bilateral ventricles, heart	NR	15% patients had a cardiac events. DVHs were negative for both events and OS on MVA.
Han 2014 [2]	RWD	I–III	60–85.5Gy in 2–3.8Gy #*	NR	3DCRT	100	“CVD”, HTN	**Primary:** OS	D33≤66Gy, D66 ≤45Gy, D100 ≤40Gy	Dmax, Vx in 5Gy increments	NR	PA	RTOG	DVHs positive for OS on MVA.
Tucker 2016 [3]	RWD	III	60–76Gy in 1.8–2Gy#*	24.0	3DCRT (41%) IMRT (49%) IMPT (10%)	468	None	**Primary:** OS	NR	Dmean, V5, V30	16.6	Heart	NR	DVHs negative for OS on MVA.
Speirs 2017 [4]	RWD	II–III	50–84.9Gy in 1.5–2.8Gy #*	14.5	3DCRT (60%) IMRT (40%)	322	None	**Primary:** Cardiac events **Others:** OS	NR	Dmean, Dmax, Vx in 5Gy increments	2.6 (3DCRT) 1.8 (IMRT)	Heart	Wheatley	24% patients had a grade 3 event. DVHs positive for OS on MVA.
Vivekanandan 2017 [5]	Trial	II–III	63–73Gy in 30#*	25.0	3DCRT (96%) VMAT (4%)	78	Pre-existing ECG only	**Primary:** OS **Others:** ECG change at 6 months	D100 <45Gy, D67 <53Gy, D33 <60%	PCAs	10.3	Pericardium, AVN, 4 chambers, 4 chamber walls	Feng	DVHs positive for OS on MVA. 38% patients had ECG changes. DVHs negative for ECG changes on MVA.
Dess 2017 [6]	Trial	II–III	70	23.0	3DCRT (97%) IMRT (3%)	125	HTN, DM, "Pre-existing disease"	**Primary:** G3+ events **Others:** G2+ events, OS	V40 <100%, V65 <33%	Dmean, V5, V30, V50	11.0	Heart	Feng	15% patients had a cardiac event. DVHs positive on MVA. DVHs positive for OS on MVA.
Wang 2017 [7]	Trial	III	70–90Gy in 23-45#*	NR	3DCRT	112	CAD	**Primary:** Symptomatic events **Others:** OS, PCE	V40 <100%, LV V40 <100%	Dmean, V30, V5, LV V30, LV V5	12.0	Heart	Feng	23% patients had a cardiac event. DVHs positive on MVA. DVHs negative for OS on MVA.
Wang 2017 [8]	Trial	III	70–90Gy in 23-45#*	NR	3DCRT	112	CAD	**Primary:** PCE, ACS, arrhyth **Others:** OS	V40 <100%, LV V40 <100%	Dmean, V5, V30, V60	12.0	Feng for chambers, LAD + automated pericardium	Feng	8%, 7%, 11% of patients had a PCE, ACS, and arrhythmia events. DVHs positive on UVA (no MVA). DVHs negative for OS on MVA.
Johnson 2017 [9]	RWD	II–III	60–74Gy in 1.8–2Gy #*	16.8	3DCRT (38%) IMRT (62%)	86	None	**Primary:** OS	None	V5, V30	NR	Heart	NR	DVHs positive for OS on MVA.
Ma 2017 [10]	RWD	I–III	60–76Gy in 30–38#*	16.9	3DCRT (89%) IMRT (11%)	141	HTN	**Primary:** OS	V30 ≤40-60%, V40 ≤ 30-50%	Dmean, Dmax, Vx in 5Gy increments	5.2	Heart, PA	RTOG	DVHs positive for OS on MVA.
Stam 2017 [11]	RWD	II–III	70.1	55.0	IMRT	469	None	**Primary:** OS	Dmax ≤40Gy, D66 ≤50Gy, D33 ≤66Gy	V0.5, V1, V2, V3, V4 and Vx in 5Gy increments & equivalent uniform dose	N/A	Heart	Feng	DVHs positive for OS on MVA.
Guberina 2017 [12]	Trial	III	45Gy in 30# +/- 26Gy in 13#*	72.0	3DCRT	155	None	**Primary:** OS	NR	Dmean, V5	13.8-17.4	Heart	Feng	DVHs negative for OS on MVA.
McWilliam 2017 [13]	RWD	NR	55Gy / 20#	NR	3DCRT (NR) /IMRT (NR)	1101 / 89 (SABR)	None	**Primary:** OS	NR	Dmax	NR	Heart base region (not delineated)	N/A	Voxel-based analysis with positive MVA for a dose region approximating to the heart base
Yegya-Raman 2018 [14]	RWD	II–IV	60–66Gy in 1.8–2Gy #*	47.4	3DCRT (64%) 3DCRT +IMRT (16%) IMRT (19%)	140	CAD, “significant arrhythmia, pericardial disease, HF without CAD, valve disease	**Primary:** Symptomatic events **Others:** OS	V40 <100%, MHD <26Gy as per QUANTEC 2010	Dmean, V5, V30, V50	15.8	4x chambers, LAD	Feng	29% of patients had an event. DVHs positive on MVA. DVHs negative for OS on MVA.
Lee 2018 [15]	RWD	III	55–70Gy in 1.8–2.75Gy #*	17.6	3DCRT (42%) IMRT/ VMAT (58%)	120	DM, IHD (with definition)	**Primary:** MI **Others:** OS	“QUANTEC” (from 2010)	Dmean, V5, V25, V30, V40, V50, D30	12.6	Heart	RTOG	MI occurred in 4%. DVHs negative for MI on MVA. DVHs positive for OS on MVA.
Lee 2019 [16]	RWD	I–III	50–54Gy in 1.8–2Gy #*	36.6	3DCRT (70%) IMRT/ VMAT (30%)	43	DM, IHD (with definition)	**Primary:** MI **Others:** OS	Dmean ≤40Gy, V60 ≤33%, V40 ≤80%, V45 ≤60%, V60 ≤33%	Dmean, V5, V25, V30, V40, V50, D30	9.4	Heart	NR	No MI events occurred. DVHs negative for OS on MVA.
Borkenhagen 2019 [17]	RWD	I–IV	44–69Gy in 14–34#*	13.2	NR	76	DM, PVD, "Previous cardiac disease"	**Primary:** Cardiac events **Others:** OS	NR	Dmean, Dmax, V30, V45	N/A	Atria, ventricles, pericardium, coronary space	Wheatley	7%, 21%, 1% of patients had atrial arrhythmia effusions, valve disease. DVHs positive on MVA. DVHs negative for OS on MVA.
Atkins 2019 [18]	RWD	II–III	50–66Gy in 1.8–2Gy #*	20.4	3DCRT (76%) IMRT (24%)	748	HTN, Lipids, DM, VTE, arrhythmia, valve disease, PVD, stroke, CAD, MI, HF **Primary:** MACE	**Others:**	G3+ events, OS None pre-2008, V30 <50%, V45 <40%, V60 <20% thereafter	Dmean, V5, V30	12.3	Heart	Feng	10% of patients had MACE. DVHs positive on MVA. DVHs positive for OS on MVA.
Hotca 2019 [19]	RWD	III	50–80Gy in 1.8–2Gy #*	20.0	IMRT	155	DM, DL, HTN, cardiac disease (list included)	**Primary:** ECG Changes	NR	PCAs, Dmin, Dmean, Dmax	NR	Chambers, great vessels	Feng	66%, 35% and 67% patients had an ECG change that was arrhythmic, ischaemic/pericarditic, or non-specific. DVHs positive on UVA (MVA not done).
McWilliam 2020 [20]	RWD	III	55Gy/20#	NR	3DCRT (NR) /IMRT (NR)	978	None	**Primary:** OS	NR	Dmax	NR	Heart base region (not delineated)	N/A	Voxel-based analysis with positive MVA for a dose region approximating to the heart base
McWilliam 2020 [21]	RWD	III	55Gy/20#	NR	3DCRT (NR) /IMRT (NR)	648	None	**Primary:** OS	NR	Dmax	NR	Right atrium surface (not delineated)	N/A	Surface dose mapping analysis with positive MVA for a dose region approximating to the right atrium
Thor 2020 [22]	Trial	III	60 or 74Gy in 2Gy #*	24.0	3DCRT (52) /IMRT (48)	306 training /131 validation	None	**Primary:** OS	Heart V33 <60Gy, V66 <45Gy, V100 <40Gy	Dmean, Dmax, Dmin, MOHX% Dmean, Dmax, and in 5Gy increments	NR	Heart, pericardium, bilateral atria, bilateral ventricles	RTOG	Model combining atria D45, lung Dmean, pericardium MOH55, and ventricles MOH5.
Jang 2020 [23]	RWD	III	50 – 72 Gy/25 – 36#	27.5	3DCRT (NR) /IMRT (NR)	258	HTN, DM, CV disease	**Primary:** OS	NR	Mean, V5, V10, V20, V30, V40, V50, and V60	NR	WH, RA, LA, RV, LV	NR	MVA positive for LV in patients with cardiac history only
Vivekanandan 2021 [24]	RWD	I–III	69	38.0	3DCRT (75%) VMAT (25%)	64	"Baseline cardiac comorbidity"	**Primary:** OS	NR	Dmean, Vx in multiple thresholds	7.6	Heart, LA	Feng	DVHs positive for OS on MVA.
Sheperd 2021 [25]	RWD	I–III	45–70Gy in 1.8–2Gy#*	64.0	3DCRT (30%) IMRT (70%)	284	None	**Primary:** OS	V30 ≤50%	Dmean, Dmin, Dmax plus Vx in 5Gy increments	11.2	Heart	RTOG	DVHs positive for OS on MVA.
Xu 2021 [26]	Trial	II–IV	60–74Gy in 2Gy # *	26.2	IMRT (61%)/Protons (39%)	225	“Pre-existing heart disease”	**Primary:** Cardiac events **Others:** OS	V30 <50%, V45 <40%, V60 <20%	Dmean, Dmax, Vx in 5Gy increments	12.0	Heart, pericardium, chambers	RTOG	25% of patients had a cardiac event. DVHs not positive for events on UVA. (No MVA). DVHs not positive for OS on UVA. (No MVA).
Atkins 2021 [27]	RWD	II–III	50–66Gy in 1.8–2Gy #*	NR	3DCRT (76%) IMRT (24%)	701	HTN, Lipids, DM, VTE, arrhythmia, valve disease, PVD, stroke, CAD, MI, HF **Primary:** MACE	**Others:**	G3+ events, OS None pre-2008, V30 <50%, V45 <40%, V60 <20% thereafter	Dmean, Dmax, Vx in 5Gy incre(26)ments	12.3	Chambers & coronaries	Feng	DVHs positive for both MACE and OS.
Niska 2021 [28]	RWD	III	43.1–74Gy in 1.8–2Gy #*	18.0	3DCRT (41%)/IMRT (59%)	119	None	**Primary:** OS	Dmax <62Gy, Dmean <26Gy, V30 <46%, V40 <33%	Vx in 5Gy increments	NR	Heart	NR	DVHs positive for OS on MVA.
McKenzie 2022 [29]	Trial	III	60Gy in 30# or 74Gy in 37#*	22.9	3DCRT (54%) IMRT (46%)	439	None	**Primary:** OS	None	Dmean, LAD V15	NR	Heart, LAD	Feng	DVHs positive for OS.
Yu 2022 [30]	RWD	III	50–72Gy in 20–36# (IMPT) or 45–66Gy in 15–33#*	25.5	IMPT (21%) IMRT/ VMAT (79%)	163	HTN, CAD, DM	**Primary:** Cardiac events	NR	Dmean, Dmax, V5, V10, V20, V30, V40,	3 (IMPT)/ 9 (IMRT)	Heart	NR	12% of patients had an event after IMRT/VMAT, 0% after IMPT.
Cho 2022 [31]	RWD	III	60–66Gy in 2–2.4Gy #*	45.0	3DCRT (83%)/3DCRT +IMRT (3%) /IMRT (14%)	133	Pre-existing heart disease (list included)	**Primary:** G2+ cardiac events	None mandatory	Dmean, V5, V30, V50	8.3	Heart, LV wall	NR	32% patients had a cardiac event. DVHs positive for events on MVA.
Craddock 2022 [32]	Trial	II–III	60 – 74 Gy/30 – 37#	NR	3DCRT (NR) /IMRT (NR)	172	LVEF	**Primary:** OS	V40 ≤50%	Dmax	NR	Heart base region (not delineated)	N/A	Voxel-based analysis with positive MVA for a dose region approximating to the heart base
Kim 2022 [33]	RWD	I–III	60-64.5Gy in 30#*	36.2	3DCRT (34%) IMRT (66%)	321	BMI, DM, AF, valve disease, CAD, CHB, stroke, PVD, CAC, alcohol	**Primary:** Cardiac events **Others:** OS	Dmean <45Gy	Dmean, Dmax, Vx in 5Gy increments	12.3	Chambers, coronaries, conduction nodes	Feng, Loap	5% of patients had AF. 2% had non-AF events. DVHs positive for AF on MVA. DVHs positive for OS on MVA.
Banfill 2022 [34]	RWD	I–IV	>45Gy in 20#*	NR	NR	967	HTN, IHD, Myocardial, Pericardial & Valve disease, Arrhythmia (list included)	**Primary:** Cardiac death	V30 <40%, V40 <30% (from 2015)	Dmean, V5, V30, V50,	12.8	Heart	UK SABR Consortium Guidance	DVHs positive for OS on MVA.
No 2023 [35]	RWD	IIB–IIIC	NR	74	3DCRT (<1%)/ IMRT (25%)/ VMAT (75%)	233	DM, HTN, previous CV event	**Primary:** Myocardial, conductive, constrictive, valvular	NR	Mean, V7, V15, V27	9.3	WH, LV, LMC, RCA, LCX, LAD, Total left coronary arteries	Duane	22% patients had a cardiac event. Total left associated with cardiac events on MVA
McWilliam 2023 [36]	Trial	III	60 – 74 Gy/30 – 37#	24	3DCRT (NR) /IMRT (NR)	458	None	**Primary:** OS	V40 <100%, V45 <66%, V60 <33%	V5, V30	NR	Heart and heart base region (not delineated)	NR	Voxel-based analysis with positive MVA for a dose region approximating to the heart base
Yegya-Raman 2023 [37]	RWD	II–IV	66Gy/33#	29.9	3DCRT (11%)/IMRT (36%)/ proton (57%)	187 / 140	Cardiac comorbidity, CAD	**Primary:** Death without progression **Secondary:** OS	MHD <26Gy	Mean, V5, V30, V50	8.1	Heart,	Feng	Ventricle metrics MVA positive for DWP and OS.
Yegya-Raman 2024 [38]	RWD	II–IIIC	66–70 Gy/33–35#	39.6	3DCRT (6%)/IMRT (59%)/ proton (35%)	335	HTN, DM, Lipids, previous atrial arrhythmia	**Primary:** MACE **Secondary:** Cardiac events, OS	MHD <20Gy, V50 <25%	Mean, Dmin, Dmax, V5-70	8.7	Heart, LV, LAD	Feng	10.4% patients had a MACE. MVA positive for OS only, for MHD and LAD V15
Olloni 2024 [39]	RWD	NR	24–33 #	NR	3DCRT (9%) / IMRT (41%)/ VMAT (50%)	644	Arrhythmia and “baseline heart disease”	**Primary:** OS	NR	V2 – V70 (2Gy intervals)	11.3	WH, RA, LA, RV, LV, LMC, LCX, LAD, RCA	NR (auto)	MVA positive for OS (2 x PCAs)
Walls 2024 [40]	RWD	I–III	55–84 Gy/20–42#	NR	3DCRT (29%)/IMRT (20%)/ VMAT (51%)	478	HTN, DM, Lipids, previous MI, previous HF, previous arrhythmia	**Primary:** OS **Secondary:** MI, HF, arrhythmia	MHD <26Gy	Dmax	NR	Heart base region	Christie Definition	17% patients had a cardiac event. MVA positive for both endpoints
Walls 2024 [41]	RWD	I–III	55Gy/20#	NR	3DCRT (20%)/ IMRT (30%)/ VMAT (50%)	420	HTN, DM, Lipids, previous MI, previous HF, previous arrhythmia	**Primary:** AF	MHD <26Gy	Mean, V5, V55	NR	Pulmonary veins	Walls	6% patients developed AF. MVA positive
Atkins 2024 [42]	RWD	II–III	50–66Gy in 1.8–2Gy #*	20.4	3DCRT (76%) IMRT (24%)	748	HTN, Lipids, DM, VTE, arrhythmia, valve disease, PVD, stroke, CAD, MI, HF	**Primary:** G3 arrhythmia subtypes	None pre-2008, V30 <50%, V45 <40%, V60 <20% thereafter	Dmean, Dmax, Vx in 5Gy increments	12.3	Chambers, coronaries, PVs, nodes	Feng	17% experienced ≥1 G3 arrhythmia. On MVA analyses, PVs associated with AF and SVT, LMC with VT/asystole, LCX with atrial flutter, and the RCA with bradyarrhythmia

*(N number of patients, EQD2 equivalent dose in 2 Gy fractions, NR not reported, DVH dose-volume histogram, MHD mean heart dose, 3DCRT three-dimensional conformal radiotherapy, IMRT intensity-modulated radiotherapy, HTN hypertension, DM diabetes mellitus, OS overall survival, MVA multivariate analysis, PCE pericardial event, ACS acute coronary syndrome, LAD left anterior descending coronary artery, UVA univariate analysis, DL dyslipidaemia, VTE venous thromboembolism, PVD peripheral vascular disease, CAD coronary artery disease, MI myocardial infarction, HF heart failure, MACE major adverse cardiac event, RTOG Radiation Therapy Oncology Group, IMPT intensity-modulated proton therapy, ECG electrocardiogram, AVN atrioventricular node, CVD cardiovascular disease, PA pulmonary artery, LV left ventricle, RV right ventricle, LA left atrium, RA right atrium, RCA right coronary artery, LMC left main stem coronary artery, LCX left circumflex coronary artery, IHD ischaemic heart disease, BMI body mass index, AF atrial fibrillation, CHB complete heart block, CAC coronary artery calcification, SABR stereotactic ablative radiotherapy, DWP death without progression, VBA voxel-based analysis, MOHX%[Gy] mean of the hottest x% of the volume, in Grey; LVEF = left ventricular ejection fraction)*

(* = EQD2 not reported, so physical prescription provided).

The figures within the PDF version of the original article were inadvertently published in black and white; they have been replaced with the original, colour versions provided by the authors at the time of submission.

The original article has been corrected.
