# Supplementary material for: Cardiotoxicity following thoracic radiotherapy for lung cancer
Source: Br J Cancer. 2024 Nov 6;132(4):311–25. doi: 10.1038/s41416-024-02888-0 (PMC11833127; doi:10.1038/s41416-024-02888-0)
Supplement: Supplementary file 1 — Supplementary Material [file 41416_2024_2888_MOESM1_ESM.docx]

**Supplementary Table 1.** Variations in superior heart border according to different atlases, in order of decreasing craniocaudal height

| **Guideline** | **Superior Heart Border** |
| --- | --- |
| **Feng Atlas**^1^ | Just inferior to the left pulmonary artery |
| **UK SABR Consortium Guidelines**^2^ | The CT slice where the pulmonary trunk and right pulmonary artery are seen as separate structures |
| **Radiation Therapy Oncology Group**^3^ | One slice below where pulmonary artery trunk passes the  midline |
| **Amsterdam Maastricht Atlas**^4^ | Superior aspect of left atrium, with great vessels excluded |

**Supplementary Table 2.** Studies assessing the utility of circulating biomarkers of radiation cardiotoxicity in lung cancer populations.

| **Article** | **Patient**  **Numbers** | **Radiotherapy Details** | **Cardiac**  **Dosimetry**  **Details** | **Time-Points** | **Markers** | **Conclusion** |
| --- | --- | --- | --- | --- | --- | --- |
| Demissei 2019^5^ | n=13 lung cancer, n=14 oesophagus | 50.4–60Gy in  1.8–2Gy # | MHD 8.4Gy  V5 34.5%  V30 7.9% | 1. “Pre-radiotherapy” 2. 20 days post-RT (median) | hsTnT | No correlation of markers with change in echo parameters |
|  |  |  |  |  | NT-proBNP |  |
|  |  |  |  |  | PGF |  |
|  |  |  |  |  | GDF-15 |  |
| Kozak  2008^6^ | n=18 NSCLC, n=4 SCLC, n=7 for oesophageal, n=1 thymic carcinoma | 36–75Gy in  1.8–2.5Gy # | MHD 12.7Gy  Dmax 50.3Gy  V5 63.4%  V10 44.8%  V20 22.1%  V30 11.9%  V40 4.3% | 1. 0-8 days before RT 2. 8-10 days on RT 3. 42 days post-RT | TnT | No positive signals |
|  |  |  |  |  | Serum CK-MB |  |
|  |  |  |  |  | NT-proBNP |  |
| Kuo  2015^7^ |  |  |  |  | CRP | Rise in SAAF does not correlate to eventual cardiovascular events |
|  |  |  |  |  | SAAF |  |
|  |  |  |  |  | VCAM-1 |  |
|  |  |  |  |  | ICAM |  |
|  |  |  |  |  | Interleukins |  |
|  |  |  |  |  | GCSF |  |
|  |  |  |  |  | TNF |  |
|  |  |  |  |  | FGF |  |
|  |  |  |  |  | PGF |  |
|  |  |  |  |  | VEGF |  |
|  |  |  |  |  | Soluble VEGF Receptor-1 |  |
| Gomez  2014^8^ | n= 5 NSCLC, n=16 mesothelioma, n=2 thymoma, n=2 others  with estimated MHD >20Gy | 45–70Gy in 1.8–2.3Gy # | MHD 26.5Gy  V40 23.4% | 1. After Fraction 1 2. Final fraction 3. 1-2 months post-RT | TnT | BNP increases correlated with irradiated heart volume |
|  |  |  |  |  | BNP |  |
| Serrano 2016^9^ | n=10 lung cancer, n=6 breast with estimated Dmax >10Gy | 9.4Gy | Dmax 64.6Gy | 1. “Baseline” 2. After fraction 1 3. Mid-point RT 4. Final fraction 5. 6 months post-RT | TnI | None of the markers tested have utility |
|  |  |  |  |  | BNP |  |
|  |  |  |  |  | Galectin-3 |  |
| Hawkins 2019^10^ | n=63 lung cancer | Mean target dose 74.4Gy EQD2 | MHD 13.7Gy | 1. <7 days prior to fraction 1 | 14 x mi-RNA | Higher levels of 5 were associated with increased G3+ cardiotoxicity  Higher levels of 9 were associated with decreased risk |
| Canada 2020^11^ | n=15 lung, n=10 breast with V5 ≥10% | NR | NR | 1. 1.8 years post-RT (median) | hsCRP | hsCRP reflected echo findings but no DVH data available |
| Xu 2021^12^ | n=225 NSCLC | 60–74Gy in  2Gy # | MHD 12.0Gy | 1. <2 months pre-RT 2. 2–3 during RT 3. 4–12 weeks post-RT | hsTnT | hsTnT likely to have utility |
| Kemal 2016^13^ | n=22 lung, n=13 breast | 40–60Gy in  1.8-2Gy # | NR | 1. Baseline 2. Last Day of RT 3. One month after RT | BNP | No clear link between cardiotoxicity and cardiac biomarkers |
|  |  |  |  |  | TnT |  |

*(MHD = mean heart dose; hsTnT = high-sensitivity troponin T; NT-proBNP = N-terminal pro-BNP; PGF = placental growth factor; GDF-15 = growth differentiation factor 15; CK-MB = creatinine kinase MB; NSCLC = non-small cell lung cancer; SCLC = small cell lung cancer; CRP = C-reactive protein; SAAF = serum ayloud A factor; VCAM-1 = vascular adhesion molecule 1; ICAM = intracellular adhesion molecule; GCSF = granulocyte colony stimulating factor; TNF = tissue necrosis factor; FGF = basic fibroblast growth factor; VEGF = vascular endothelial growth factor; EQD2 = equivalent dose in 2Gy fractions)*

**Supplementary Table 3.** Characteristics of the first prospective translational studies in radiation cardiotoxicity focussed on patients with lung cancer

| **NCT ID** | **Abbreviation** | **Location** | **Design** | **Patients** | **Stage** | **Treatment** | **Parameters** |
| --- | --- | --- | --- | --- | --- | --- | --- |
| **04305613** | CLARITY | USA | Multicentre, observational | 221 | II–III | (chemo)RT +/- durvalumab | PET-CT, TTE, bloods, QOLS |
| **03645317** | ACcoLade | UK | Two centres, observational | 137 | I–III | (chemo)RT  or SABR | Cardiac CT, TTE, ECG, bloods |
| **04867564** | N/A | Poland | Single centre, observational | 100 | I–III | (chemo)RT | TTE |
| **05252065** | N/A | China | Single centre, observational | 40 | III | (chemo)RT | CMR, TTE, bloods |

(RT = radiotherapy; SABR = stereotactic ablative radiotherapy; TTE = transthoracic echocardiogram; ECG = electrocardiogram; PET = positron emission tomography; CT = computed tomography; QOLS = quality of life scoring; CMRI = cardiac magnetic resonance imaging)

**REFERENCES**

1. Feng M, Moran JM, Koelling T, et al. Development and validation of a heart atlas to study cardiac exposure to radiation following treatment for breast cancer. *Int J Radiat Oncol Biol Phys*. 2011;79(1):10-18. doi:10.1016/J.IJROBP.2009.10.058

2. UK SABR Consortium. *UK SABR Consortium Guidelines Version 6*.; 2019. Accessed May 19, 2024. https://www.sabr.org.uk/wp-content/uploads/2019/04/SABRconsortium-guidelines-2019-v6.1.0.pdf

3. Radiation Thoracic Oncology Group. Atlases for Organs at Risk (OARs) in Thoracic Radiation Therapy. Published online December 1, 2011. Accessed May 12, 2024. https://www.eviq.org.au/getmedia/a4c012a8-d6a7-4d87-93f7-d3f465b49889/RTOG-heart-contouring-atlas.pdf.aspx?ext=.pdf

4. National Radiotherapy Group. *Amsterdam-Maastricht OAR Atlas*.; 2016.

5. Demissei BG, Freedman G, Feigenberg SJ, et al. Early Changes in Cardiovascular Biomarkers with Contemporary Thoracic Radiation Therapy for Breast Cancer, Lung Cancer, and Lymphoma. *Int J Radiat Oncol Biol Phys*. 2019;103(4):851-860. doi:10.1016/J.IJROBP.2018.11.013

6. Kozak KR, Hong TS, Sluss PM, et al. Cardiac blood biomarkers in patients receiving thoracic (chemo)radiation. *Lung Cancer*. 2008;62(3):351-355. doi:10.1016/J.LUNGCAN.2008.03.024

7. Kuo AH, Ancukiewicz M, Kozak KR, Yock TI, Padera TP. Cardiac and inflammatory biomarkers do not correlate with volume of heart or lung receiving radiation. *Radiat Oncol*. 2015;10(1). doi:10.1186/S13014-014-0324-1

8. Gomez DR, Yusuf SW, Munsell MF, et al. Prospective exploratory analysis of cardiac biomarkers and electrocardiogram abnormalities in patients receiving thoracic radiation therapy with high-dose heart exposure. *Journal of Thoracic Oncology*. 2014;9(10):1554-1560. doi:10.1097/JTO.0000000000000306

9. Serrano NA, Mikkelsen R, Canada J, Mezzaroma E, Weiss E, Abbate A. Biomarkers of cardiac injury in patients undergoing thoracic radiation therapy. *Int J Cardiol*. 2016;223:507-509. doi:10.1016/J.IJCARD.2016.08.263

10. Hawkins PG, Sun Y, Dess RT, et al. Circulating microRNAs as biomarkers of radiation-induced cardiac toxicity in non-small-cell lung cancer. *J Cancer Res Clin Oncol*. 2019;145(6). doi:10.1007/S00432-019-02903-5

11. Canada JM, Thomas GK, Trankle CR, et al. Increased C-reactive protein is associated with the severity of thoracic radiotherapy-induced cardiomyopathy. *Cardiooncology*. 2020;6(1). doi:10.1186/S40959-020-0058-1

12. Xu T, Meng QH, Gilchrist SC, et al. Clinical Investigation Assessment of Prognostic Value of High-Sensitivity Cardiac Troponin T for Early Prediction of Chemoradiation Therapy-Induced Cardiotoxicity in Patients with Non-Small Cell Lung Cancer: A Secondary Analysis of a Prospective Randomized Trial. *Int J Radiation Oncol Biol Phys*. 2021;111(4):2021. doi:10.1016/j.ijrobp.2021.07.035

13. Ekici K, Cakal B, Baydar O, et al. Measurement of strain imaging, troponin-I and brain natriuretic peptide levels in radiotherapy-induced cardiac injury. *UHOD - Uluslararasi Hematoloji-Onkoloji Dergisi*. 2016;26(2):75-82. doi:10.4999/uhod.161170
